# Comparative analysis of the GBLUP, emBayesB, and GWAS algorithms to predict genetic values in large yellow croaker (*Larimichthys crocea*)

**DOI:** 10.1186/s12864-016-2756-5

**Published:** 2016-06-14

**Authors:** Linsong Dong, Shijun Xiao, Qiurong Wang, Zhiyong Wang

**Affiliations:** Key Laboratory of Healthy Mariculture for the East China Sea, Ministry of Agriculture, Fisheries College, Jimei University, Xiamen, Fujian Peoples’ Republic of China

**Keywords:** Large yellow croaker, Genotyping-by-sequencing, Genomic selection, Predictive ability, Genome-wide association study

## Abstract

**Background:**

The advances of sequencing technology accelerate the development of theory of molecular quantitative genetics such as QTL mapping, genome-wide association study and genomic selection. This paper was designed to study genomic selection in large yellow croaker breeding. The aims of this study were: (i) to estimate heritability values of traits in large yellow croaker; (ii) to assess feasibility of genomic selection in the traits of growth rate and meat quality; (iii) to compare predictive accuracies affected by different algorithms and training sizes, and to find what training sizes could reach ideal accuracies; (iv) to compare results of GWAS with genomic prediction, and to assess feasibility of pre-selection of significant SNPs in genomic selection. 500 individuals were tested in the trait of body weight and body length, while 176 were tested in the percentage of n-3 highly unsaturated fatty acids (n-3HUFA) in muscle. GBLUP and emBayesB were used to perform genomic prediction.

**Results:**

Genotyping-By-Sequencing method was used to construct the libraries for the NGS sequencing and find ~30,000 SNPs. Heritability estimates were 0.604, 0.586 and 0.438 for trait of body weight, body length and n-3HUFA, respectively. The predictive abilities estimated by GBLUP showed higher than that by emBayesB in traits of body weight and body length. However, the result was just the opposite in n-3HUFA. According to fit the curve of predictive accuracy, we estimated that at least 1000 individuals in training set could reach an accuracy of 0.8 in body weight and body length. GBLUP, emBayesB and GWAS could not always find significant SNPs associated with phenotypes consistently. Significant SNPs were selected by emBayesB could obtain the largest proportions to explain total additive genetic variances.

**Conclusions:**

This research showed that genomic selection was feasible in large yellow croaker breeding. We suggest doing a test before deciding to use which algorithm in specific trait in genomic prediction. We estimated required training sizes to reach ideal predictive accuracies and assessed feasibility of pre-selection of SNPs successfully. Because of high mortality rate of fish and high cost in genomic sequencing, genomic selection may be more suitable for applying on some traits which cannot be measured on candidates directly.

## Background

With the advent of next generation sequencing technologies, plants and animals can be genotyped for thousands of single nucleotide polymorphisms (SNPs) at one time. Sequencing technologies accelerate the development of theory of molecular quantitative genetics. Quantitative trait loci (QTL) mapping and genome-wide association study (GWAS) have been considered as new methods applied in breeding programs. Quantitative traits, however, were verified to be affected by many genes termed as QTLs [1]. In GWAS, each QTL is identified based on a significance test. As a result, many QTLs will be ignored because most QTLs have smaller effects and can’t reach the significant levels [2, 3]. In order to avoid the above defect, a new method termed as genomic selection (GS) was proposed by Meuwissen et al. [4]. Genomic selection uses entire genomic data to explain observed phenotypic variation, but not selects single locus based on a significance test. With high density markers, each QTL can be highly in linkage disequilibrium (LD) with at least one marker [5]. Due to the advantages of high accuracy of prediction and reduction of generation interval [6], genomic selection has been widely used in dairy cattle [7–13] and has been studied in other species [14–21]. Compared with livestock and plant breeding, genomic selection is relatively late to be applied in aquatic breeding [22]. Sonesson et al. [23] have studied genomic selection in aquaculture breeding programs by using simulation data but not real data. Recently, an experiment on genomic selection in Atlantic salmon was studied by Hsin-Yuan et al. [24]. This paper was designed to discuss the feasibility of genomic selection applying in large yellow croaker breeding.

Large yellow croaker (*Larimichthys crocea*) is one of the most important commercial marine fish species in southeast China and Eastern Asia [25]. However, the genetic diversity of large yellow croaker is seriously lost because of over-fishing and environmental degradation [26]. In addition, the fishing technology reserving larger and abandoning smaller individuals gives rise to negative selection for large yellow croaker. Therefore, a good breeding technique is necessary for this species. In traditional animal breeding, genetic values are predicted from the phenotypic data of individuals and their relatives. This algorithm is termed as best linear unbiased prediction (BLUP) [27]. However, BLUP cannot estimate Mendelian sampling term very well [28]. Using genome-wide SNP genotypes may be a better choice to obtain more accurate relationship among relatives. Nielsen et al. [29] have used simulation method and Hsin-Yuan et al. [24] have used real data to compare the accuracies for genomic estimated breeding values (GEBV) with traditional BLUP estimated breeding values (BLUPEBV), and have suggested that accuracy for GEBV was higher than that for BLUPEBV in aquaculture. Therefore, it may be a trend to apply marker-assisted selection (MAS) in fish breeding programs. Especially for some traits, such as meat quality and disease resistance, which cannot be measured on candidates directly, are more suitable to use MAS schemes for breeding.

Various algorithms are used to predict GEBV in genomic selection, including Genomic BLUP (GBLUP) [30] and Bayesian methods [4]. GBLUP was deduced by VanRaden by using genomic relationship matrix (G matrix) to obtain GEBV directly. Another algorithm termed as RR-BLUP (ridge-regression BLUP) can obtain the same results as GBLUP by calculating SNP effects firstly, which was firstly proposed by Meuwissen et al. [4]. The prior distribution of GBLUP algorithm assumes an equal variance across each locus, which is not an accurate assumption when number of QTLs is small [31]. Nevertheless, it is closer to reality if many QTLs exist in the genome. Another assumption is that there are many loci with no variance and a few loci with their own variances [4]. This algorithm is termed as BayesB, which has a mixture of prior distribution. The GEBV are estimated based on MCMC (Monte Carlo Markov Chain) technology in BayesB, which needs much more computing time. The reason is that the prior distribution of markers is proposed in term of variances but not of effects. Therefore, in order to save the computing time, Meuwissen et al. [32] proposed to use a mixture of a distribution with zero effects and an exponential distribution as a prior for the marker effects:

1 $$ \pi (g)=\left\{{}_{\left(1-\gamma \right)\kern5.25em for\ g = 0}^{\raisebox{1ex}{$1$}\!\left/ \!\raisebox{-1ex}{$2$}\right.\ast \gamma \lambda exp\left(-\lambda \left|g\right|\right)\kern0.5em for\ g\ne 0}\right. $$

where *γ* is the proportion of makers existing effects, and *λ* is the parameter of exponential distribution. This algorithm termed as fast BayesB or iterated conditional expectation (ICE) is not based on MCMC technology, therefore the computing speed is several orders of magnitude faster than MCMC based BayesB. On the basis of the study of ICE, Shepherd et al. [33] developed an algorithm (named emBayesB) by combining expectation-maximization (EM) algorithms with fBayesB. Besides the fast computational speed and relatively high estimation accuracy, emBayesB has other advantages: (i) the algorithm can adjust the value of proportion that SNPs are in LD with QTLs in the calculating process; (ii) heritability, which is set beforehand, hardly affects the estimation results even if the heritability deviates from the actual situation significantly.

Predictive accuracy of GEBV is one of the most important indicators in genomic selection, which has been studied by various methods based on real or simulated data [4, 34–37]. It is affected by many factors, such as training sizes, trait heritability, number of QTLs, and also by marker density and statistical methods. This research would estimate trait heritability and assesse the predictive abilities via cross-validation, and compare predictive abilities within various training records and two algorithms (GBLUP and emBayesB). Combined with the formula for predictive accuracy in genomic selection [34], we would predict the training sizes required to reach ideal predictive accuracies. We would also compare results of GWAS with genomic prediction, and assess feasibility of pre-selection of significant SNPs in genomic selection.

## Methods

### Materials

The experimental materials were large yellow croaker. All fish were reared in a breeding nucleus farm named ‘Jinling Aquaculture Science and Technology Co. Ltd.’ in Ningde City, Fujian Province, P.R.China. The trail was carried out in Key Laboratory of Healthy Mariculture for the East China Sea when the age of fish was two years old. All fish were injected the hormone named Luteinizing hormone releasing hormone A3 (LRH-A3) simultaneously. Approximately 36 h after injection the LRH-A3, all fish would release sperms or eggs almost at the same time, so all progenies which were used as experimental materials had the same age. Three quantitative traits were tested: body weight (BW), body length (BL) and percentage of n-3 highly unsaturated fatty acids (n-3HUFA) in muscle. Growth rate and meat quality are the most important economic traits in large yellow croaker, so the three traits were chosen for research. BW and BL data was derived from the live body directly. n-3HUFA data, however, must be measured by dissection. 500 individuals were tested on traits of BW and BL while 176 individuals sampled randomly from the 500 individuals were tested on trait of n-3HUFA, which would be used as the experimental materials in this research. The parameters of the three traits were shown in Table 1.

**Table 1:** Statistical results of phenotypic data for three quantitative traits

Trait	Male			Female		
	Number	Mean^a^	Standard deviation^a^	Number	Mean	Standard deviation
Body weight	237	202.22	77.15	263	247.41	99.96
Body length	237	227.19	25.19	263	234.85	29.04
n-3HUFA	61	23.50	4.22	115	24.39	4.78

^a^The unit was gram (g) for BW, millimeter (mm) for BL and percentage (%) for n-3HUFA

### Library preparation and sequencing

Fin samples of all 500 fish shown in Table 1 were collected for genotyping. To detect whole-genomic SNP markers for all fish, *Eco*RI and *Nla*III based on Genotyping-By-Sequencing (GBS) method were used to construct the libraries for the NGS sequencing (had not been published). Briefly, genomic DNA of each fish individual was incubated at 37 °C with *Eco*RI and *Nla*III (New England Biolabs, NEB), CutSmart™ buffer and MilliQ water. Digestion reactions were heat-inactivated at 65 °C for 20 min and the reaction system was held in 8 °C. The digested DNA was ligated to adapter sequences with CutSmart™ buffer, ATP, T4 DNA ligase, adapter mix and MilliQ water at 16 °C. Restriction-ligation reaction was also heat-inactivated at 65 °C for 20 min and the reaction system was held in 8 °C. The PCR reaction was performed using diluted restriction-ligation samples, dNTP, Taq DNA polymerase (NEB) and IlluminaF primer and indexing primer containing barcodes. The PCR productions were separated by 8 % PAGE. Fragments with 150 ~ 350 bp (with indexes and adaptors) in size were isolated by using a Gel Extraction Kit (Qiagen), which was diluted for sequencing. Then, pair-end sequencing was performed upon the selected libaries by using an Illumina high-throughput sequencing platform (Illumina, Inc; San Diego, CA; USA).

### SNP calling and imputation

**Fig. 1:**
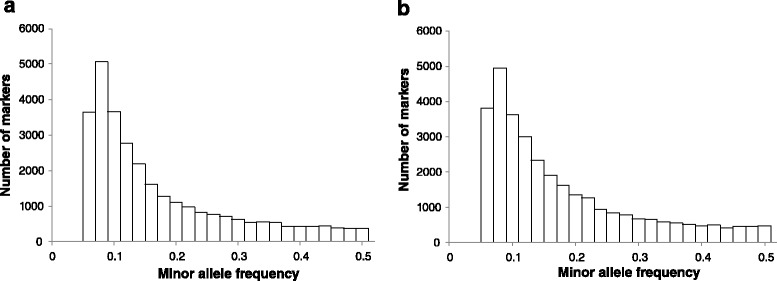
Distribution of minor allele frequency (MAF) for three traits. The left figure (**a**) showed the distribution of MAF for body weight and body length and the right figure (**b**) showed that for percentage of n-3 highly unsaturated fatty acids

The raw sequencing reads were quality checked by FastQC [38]. The reads were then quality filtered by the following steps: (1) adaptor sequences were removed from the raw reads; (2) the reads with the ratio of ambiguous ‘N’ bases greater than 5 % were filtered; (3) deleted the continuous base windows of 5 bp that the average quality smaller than 20 at two ends of reads; (4) removed short reads with a length below 50 bp. The cleaned reads were mapped to large yellow croaker reference genome sequence [39] by BWA version 0.7.10 [40]. The alignments files were then sorted and duplicate marked by Picard (http://picard.sourceforge.net) and applied to GATK package [41] for SNP calling. The resulting SNPs were discarded according to any of the criterions: (1) missing rate ≥20 %; (2) MAF (minor allele frequency) < 0.05; (3) significantly deviation from Hardy-Weinberg equilibrium (HWE) (*p*-value < 0.001). As a result, 29,748 SNPs were retained for BW and BL, and 32,249 SNPs were retained for n-3HUFA, and the average missing rate of markers was 11.9 %. Fig. 1 showed the distribution of minor allele frequency after filtration. Beagle Version 3.3.2 software [42] was used to impute all missing SNPs.

### Statistical methods

The following linear model is fitted to explain the composition of trait *Y*_*i*_:

2 $$ {Y}_i=\mu +se{x}_k+{\displaystyle \sum_{j=1}^p{X}_{ij}{g}_j}+{e}_i $$

where *Y*_*i*_ is the phenotypic record of individual *i* (*i* = 1, 2, …, *n*), *μ* is an overall mean, *sex*_*k*_ is the fixed effect of the *kth* sex (*k* = 1 for male or 2 for female). *X*_*i∙*_ is a *1* × *p* vector of SNP genotypes on individual *i* (The SNP genotypes are coded as 0 for genotype ‘*A*_*A*’, 1 for ‘*A*_*a*’ and 2 for ‘*a*_*a*’). *g*_*j*_ is the effect of the *j*th locus, so *g* is a *p* × *1* vector of SNP effects, and *e*_*i*_ is a residual effect. In most conditions, the value *p* is much larger than *n*. ${\displaystyle \sum_{j=1}^p{X}_{ij}{g}_j}$ can be replaced by *ĝ*_*i*_ which is the breeding value of individual *i*. If need to calculate SNP effects, the genotype codes will be standardized using the formula: ${X_{ij}}^{\prime }=\left({X}_{ij}-2{p}_j\right)/\sqrt{2{p}_j\left(1-{p}_j\right)}$, where *p*_*j*_ is the frequency of allele ‘*a*’ at locus *j*. After standardizing, the mean of genotype at locus *j* is 0 and variance is 1, so the variance of locus *j* is only decided by the effect *g*_*j*_, which was described by Meuwissen et al. in detail [32].

Two algorithms were used to calculate the effects of SNPs in this study: GBLUP [30] and emBayesB [33]. The GEBV of GBLUP are calculated by the following mixed model equation (MME):

3 $$ \left[\begin{array}{l}{{\mathrm{l}}_n}^{\prime }{\mathrm{l}}_n{{\kern1em \mathrm{l}}_{\mathrm{n}}}^{\prime}\mathrm{X}\\ {}{\mathrm{X}}^{\prime }{\mathrm{l}}_n\kern0.75em {\mathrm{X}}^{\prime}\mathrm{X}+\mathrm{K}\lambda \end{array}\right]\left[\begin{array}{l}\widehat{\mu}\\ {}\widehat{g}\end{array}\right]=\left[\begin{array}{l}{{\operatorname{l}}_n}^{\prime }y\\ {}{\mathrm{X}}^{\prime }\ y\end{array}\right] $$

where $\lambda ={\sigma_{e_i}}^2/{\sigma_{g_i}}^2=p\left(1-{h}^2\right)/{h}^2$, *h*^*2*^ is heritability of trait, *ĝ* is a vector of GEBV, and K is an inverse of G matrix in GBLUP algorithm. G matrix is calculated by the formula $\frac{\left(\mathrm{X}-P\right){\left(\mathrm{X}-P\right)}^{\prime }}{2{\displaystyle \sum {p}_i\left(1-{p}_i\right)}}$ [30], where *P* is the vector of frequency of allele ‘*a*’ in all loci, and *p*_*i*_ is the frequency of allele ‘*a*’ at locus *i*. The MME in RR-BLUP is very similar with formula (3), but is used to calculate SNP effects, and K is not the inverse of G matrix but an identity matrix, and $\lambda ={\sigma_e}^2/{\sigma_{g_i}}^2=p\left(1-{h}^2\right)/{h}^2$. The two algorithms are equivalent in predicting GEBV of candidates, so we defined both two algorithms as GBLUP in this study. *σ*_*e*_^2^ and *σ*_*g*_^2^ were estimated by the algorithm ‘REML’ (Restricted Maximum Likelihood) [43]. Another way could obtain the similar results of *σ*_*e*_^2^ and *σ*_*g*_^2^ by using the R-package ‘EMMREML’, Version 3.1 (http://mirror.bjtu.edu.cn/cran/web/packages/EMMREML/index.html). The formula (1) was still used as the prior distribution of SNP effects in emBayesB. All calculation process were written by Fortran codes (the codes of emBayesB were supplied by Shepherd et al. [33], http://www.biomedcentral.com/1471-2105/11/529/additional) and run in the server of Jimei University.

### Cross-validation

In order to reduce the errors caused by random sampling, a replicated training-testing method was used to evaluate the results of genomic prediction. Cross-validation of 20 replicates was performed in this research. 400 individuals were randomly sampled as training set and the rest 100 individuals were used as testing set in each repeat in the BW and BL experiment. The same way was used to study n-3HUFA but the number of individuals in training set and testing set was changed to 140 and 36 respectively. In each replicate, the same training set and testing set were used to perform the GBLUP and emBayesB prediction, so the results would have sufficient comparability for the two algorithms. Paired-t tests were used to test whether predictive abilities estimated by two algorithms had significant differences.

The evaluation index was predictive ability obtained by calculating the correlation between GEBV and observed values in the testing set, i.e., *r*_*(ĝ,y)*_. The relationship between predictive ability and predictive accuracy was deduced by Legarra et al. [20]:

4 $$ {r}_{\left(\widehat{g},y\right)}={r}_{\left(\widehat{g},g\right)}\ast h $$

where *y* is observed values, *ĝ* is genomic estimated breeding values (GEBV), *g* is true breeding values (TBV), and *h* is the square root of trait heritability. *r*_(*ĝ*,*g*)_ is the correlation between GEBV and TBV, which is termed as predictive accuracy. True breeding values, however, can only be observed in simulation data. Therefore, we had to substitute phenotypic data for true breeding values. Nevertheless, the predictive abilities still had comparability because *h* could be considered as a constant in the same trait and the same population.

In order to observe the changes of predictive abilities by the sizes of training set, the training sizes were also changed from the level 100 to 400 (4 levels were used with 100 as a spacing) in BW and BL experiment. Because the number of individuals was very small in n-3HUFA, the training sizes affecting predictive abilities would not be studied any longer. 20 replicates were also used in the experiment. The empirical formula for predictive accuracy was proposed by Daetwyler et al. [34]:

5 $$ {r}_{\left(\widehat{g},g\right)}=\sqrt{\frac{N{h}^2}{N{h}^2+M}} $$

where *M* is the number of independent loci affecting a trait. *h*^*2*^ is the trait heritability which can be obtained from REML algorithm. *r*_(*ĝ*,*g*)_ is derived from the formula (4), i.e., *r*_(*ĝ*,*g*)_ = *r*_(*ĝ*,*y*)_/*h*. To fit the regression equation, Olrike et al. used least-squares curve-fitting method [19]. In this study, we linearized the formula (5) to derive a linear regression equation:

6 $$ \frac{1-{r_{\left(\widehat{g},g\right)}}^2}{{r_{\left(\widehat{g},g\right)}}^2}=\frac{M}{h^2}\ast \frac{1}{N} $$

Here the assumption was that *y* was $\frac{1-{r_{\left(\widehat{g},g\right)}}^2}{{r_{\left(\widehat{g},g\right)}}^2}$ and *x* was $\frac{1}{N}$, so we obtained a linear model *y* = *kx* with no intercept, where $k=\frac{M}{h^2}$. According to formula (6), we could derive how many individuals in training set were required to reach an ideal predictive accuracy. The number of independent loci (M) could be also derived from the equation.

### Comparison of GS with GWAS

Although the objective of genomic selection is different with GWAS, it is still informative to compare the significant SNP loci which are analyzed by GWAS and calculated by GS, and it is helpful to find the best method of pre-selecting SNPs for genomic prediction. All individuals were used to perform GWAS analysis and to calculate SNP effects, i.e., 500 individuals were used in BW and BL while 176 individuals were used in n-3HUFA. A linear regression model was used to perform the GWAS analysis. Two algorithms, i.e., RRBLUP (GBLUP) and emBayesB were still used to calculate the SNP effects. We also compared the proportions of total additive genetic variances (V_A_) explained by the most significant SNPs by GWAS with that explained by the largest absolute effects calculated by GBLUP and emBayesB. The genetic variances explained by significant SNPs were also estimated by REML algorithm [43]. Theoretically, genetic variance of a locus decides the contribution to the phenotypic variance, but when the genotype has been standardized with mean 0 and variance 1, the absolute SNP effect can reflect the contribution of a locus to phenotypic variance [32].

## Results

### Heritability estimate

The heritability values estimated by algorithm REML were 0.604, 0.586 and 0.438 for trait of body weight, body length and n-3HUFA, when the number of phenotypic records was 500, 500 and 176 respectively. The results were very similar when heritability estimated by different number of phenotypic records (shown in Table 2).

**Table 2:** Heritability estimates by REML in different number of phenotypic records

Trait	No. of phenotypic records				
	100	200	300	400	140
Body weight	0.561 (0.054)	0.625 (0.034)	0.620 (0.018)	0.619 (0.013)	
Body length	0.555 (0.054)	0.607 (0.032)	0.580 (0.018)	0.596 (0.015)	
n-3HUFA					0.454 (0.026)

The results were average of 20 replicates. Standard errors of means were in the parentheses

### Predictive abilities

Table 3 showed the means and standard errors of predictive abilities estimated by GBLUP and emBayesB when training sizes were 400 for BW and BL, and 140 for n-3HUFA. The results showed that predictive abilities estimated by GBLUP were higher than that by emBayesB in trait of body weight and body length. Through the paired t-tests, the differences were extremely significant (*P* < 0.001) in body weight and significant (*P* = 0.015) in body length. However, the result was just the opposite in n-3HUFA, i.e., predictive ability estimated by emBayesB was higher than that by GBLUP, but the result of paired *t*-test did not show significant difference (*P* = 0.496) between the two algorithms.

**Table 3:** Predictive abilities of GBLUP and emBayesB for three quantitative traits

	Predictive ability (mean ± se)	
	GBLUP	emBayesB
Body weight	0.406 (0.020)	0.371 (0.020)
Body length	0.404 (0.017)	0.374 (0.013)
n-3HUFA	0.304 (0.042)	0.320 (0.032)

Predictive ability was the correlation between GEBV and observed values in testing set. Training size was 400 for BW and BL, and 140 for n-3HUFA. The results were average of 20 replicates

Table 4 showed trend of the predictive abilities with different number of individuals in training set. In general, the increase of predictive abilities accompanied with the increase of training sizes. In this study, we assumed the formula (5) was appropriate for both GBLUP and emBayesB algorithms. Combined with formula (6), we obtained the curve fitting equations of predictive accuracies, which were shown in Table 5.

**Table 4:** Predictive abilities of GBLUP and emBayesB in different number of phenotypic records

Trait	Algorithm	No. of phenotypic records			
		100	200	300	400
Body weight	GBLUP	0.315 (0.022)	0.350 (0.023)	0.384 (0.021)	0.406 (0.020)
	emBayesB	0.293 (0.019)	0.350 (0.021)	0.359 (0.020)	0.371 (0.020)
Body length	GBLUP	0.284 (0.015)	0.342 (0.019)	0.375 (0.018)	0.404 (0.017)
	emBayesB	0.268 (0.017)	0.314 (0.017)	0.356 (0.018)	0.374 (0.013)

The results were average of 20 replicates. Standard errors of means were in the parentheses

**Table 5:** Curve fitting equations of accuracies and required training sizes to reach ideal accuracies

Trait	Algorithm	Equation	Required size^a^
Body weight	GBLUP	${r}_{\left(\widehat{g},g\right)}=\sqrt{\frac{0.604\times N}{0.604\times N+371.27}}$	1093
	emBayesB	${r}_{\left(\widehat{g},g\right)}=\sqrt{\frac{0.604\times N}{0.604\times N+427.53}}$	1258
Body length	GBLUP	${r}_{\left(\widehat{g},g\right)}=\sqrt{\frac{0.586\times N}{0.586\times N+410.55}}$	1246
	emBayesB	${r}_{\left(\widehat{g},g\right)}=\sqrt{\frac{0.586\times N}{0.586\times N+478.97}}$	1453

^a^Required training size when predictive accuracy reached 0.8

### Results of GWAS, GBLUP and emBayesB

**Fig. 2:**
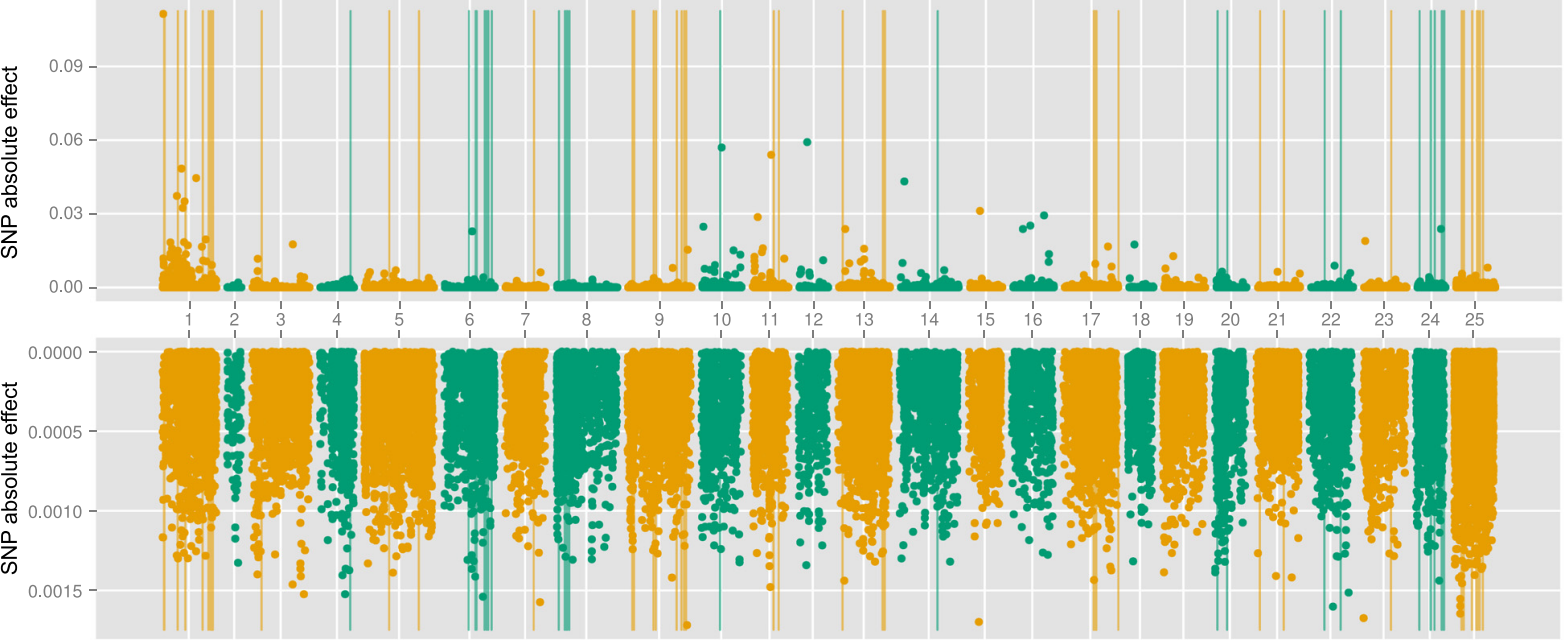
Manhattan plot of absolute SNP effects estimated by GBLUP and emBayesB for body weight. X-axis represented the chromosome number (1–24). Number 25 was not chromosome but SNPs which had not been located on specific loci in genome. The upper figure was the results of emBayesB, and the lower figure was the results of GBLUP. Vertical lines indicated the 83 significant SNP loci (*P*-value < 10^−5^) analyzed by GWAS

**Fig. 3:**
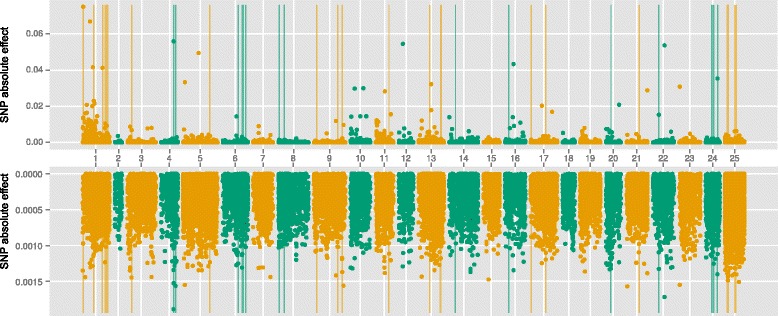
Manhattan plot of absolute SNP effects estimated by GBLUP and emBayesB for body length. X-axis represented the chromosome number (1–24). Number 25 was not chromosome but SNPs which had not been located on specific loci in genome. The upper figure was the results of emBayesB, and the lower figure was the results of GBLUP. Vertical lines indicated the 43 significant SNP loci (*P*-value < 10^−6^) analyzed by GWAS

**Fig. 4:**
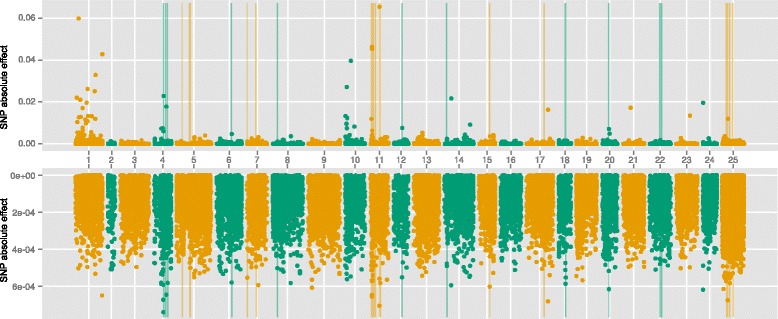
Manhattan plot of absolute SNP effects estimated by GBLUP and emBayesB for n-3HUFA. X-axis represented the chromosome number (1–24). Number 25 was not chromosome but SNPs which had not been located on specific loci in genome. The upper figure was the results of emBayesB, and the lower figure was the results of GBLUP. Vertical lines indicated the 48 significant SNP loci (*P*-value < 10^−4^) analyzed by GWAS

Figures 2, 3 and 4 showed the results of GBLUP, emBayesB and GWAS. Y-axes represented the absolution values of SNP effects estimated by GBLUP and emBayesB. The vertical lines indicated the significant SNP loci analyzed by GWAS. The results showed that significant SNPs found by GWAS tended to cluster together in some regions. By comparing the results of three algorithms, we found that three algorithms could not always found the SNPs associated with phenotypes consistently. Using body weight as an example, all algorithms could find coincident SNP loci associated with phenotypes in chromosome 1, 6, 10, 11, 13 and 24. However, some significant loci (in chromosome 4, 8 and 23) were found by GBLUP and GWAS but not found by emBayesB. Similarly, some significant loci (in chromosome 12, 15 and 16) were found by GBLUP and emBayesB but not found by GWAS. The proportions of total additive genetic variances explained by significant SNPs (or SNPs with the largest absolute effects) were shown in Table 6. We could find that the same number of significant SNPs by GWAS explained the least proportion of total additive genetic variance, and that by emBayesB could explain the largest proportion (even more than 100 % in n-3HUFA).

**Table 6:** Proportions of additive genetic variances explained by significant SNPs or SNPs with large absolute effects

	No. of SNPs^a^	Variance (Proportion)^b^			V_A_^c^
		GWAS	GBLUP	emBayesB	
Body weight	83	0.187 (30.1 %)	0.387 (62.3 %)	0.489 (78.7 %)	0.621
Body length	43	0.175 (29.7 %)	0.356 (60.3 %)	0.436 (73.9 %)	0.590
n-3HUFA	48	0.281 (63.9 %)	0.382 (86.8 %)	0.454 (103.2 %)	0.440

^a^The number of significant SNPs (or SNPs with the largest absolute effects) was selected to analyze additive genetic variance components

^b^Additive genetic variance explained by significant SNPs and the proportion in total additive genetic variance

^c^Total additive genetic variance estimated by using all SNPs

## Discussion

### Heritability estimation

Although the heritability estimated by different phenotypic records was very similar, the stability was poorer when the number of phenotypic records used to estimate heritability became smaller. For example, when 400 individuals had phenotypic records, the standard error of mean for heritability estimation was 0.013 in body weight, but the result became 0.054 when only 100 individuals had records. Therefore, we suggest using as many individuals having phenotypes as possible to estimate heritability.

### Predictive abilities

The predictive abilities by GBLUP were slightly higher than that by emBayesB in BW and BL, which was not coincident with simulation results but coincident with some real data. In most simulation results, the accuracies of Bayesian method were higher than that of GBLUP [4, 32, 33]. However, the results in some real data showed that the accuracies of GBLUP were similar to or even higher than that of Bayesian method [10, 11, 19, 44]. One point we need to pay attention to is that relatively small number (~50 or fewer) of QTLs was used in simulation study [4, 32, 33, 37]. GBLUP, however, has no advantage when number of QTLs is smaller. Therefore, we speculate more QTLs (more than 50) affecting BW and BL exist in the genome of large yellow croaker. We think another reason maybe the exponential distribution is not a suitable prior distribution in large yellow croaker. Maybe a better distribution needs to be studied in this species. However, the result was just the opposite in n-3HUFA, which may be explained by the reason that not many QTLs affecting n-3HUFA exist in genome. Another evidence could support this viewpoint in Table 6. Only 48 significant SNPs could explain most (even more than 100 %) proportion of total additive genetic variance. In view of the advantages in different algorithms, we suggest doing a test before deciding to use which algorithm to calculate marker effects and GEBV. According to results of this research, we suggest GBLUP is more suitable to perform genomic prediction in body weight and body length in large yellow croaker. The result of significance test in n-3HUFA did not show significant difference between the two algorithms. We think the reason may be relatively small training sizes were used to perform genomic prediction, which may be also the reason why the standard errors of predictive abilities in n-3HUFA were higher than that in BW and BL.

According to the predictive accuracy equations shown in Table 5, we can derive the training sizes required to reach ideal accuracies. That is to say, at least 1000 individuals are needed to reach predictive accuracy of 0.8. We think this is a very good result that only ~1000 individuals can reach a so high accuracy. The reason may be high trait heritability and the consistent rearing environment. The number of independent QTL loci (i.e., M value), was also observed from the equation, which was far more than 50, which may support our speculation in the above discussion.

### Comparison of GWAS and GS

At the present time, it is quite expensive to perform genomic selection in large yellow croaker breeding. The genotyping of a candidate by GBS still costs more than 2 to 3 broodstock, so it is necessary to compare the results of GWAS with GS, and find the best method of pre-selecting SNPs for genomic prediction. Figures 2, 3 and 4 showed significant SNPs by GWAS tend to cluster together in specific regions. The reasons may be that strong correlations exist between adjacent SNPs and single-marker analysis was used by GWAS in this study. When one SNP locus is correlated with phenotypes significantly, the adjacent locus may show similar result. Table 6 showed the significant SNPs by GWAS could explain the least proportion of total additive genetic variances, which is still caused by the clusters phenomenon. Although 83 SNP loci with *P*-value < 10^−5^ in body weight, many clustered significant SNPs just corresponded to one QTL actually.

Figures 2, 3 and 4 showed that the distributions of SNP effects were very different between GBLUP and emBayesB. The main reason is the different prior assumptions. emBayesB assumes most loci having no effects, and therefore compresses effects of most loci to near zero. GBLUP, however, assumes all loci having equal variance, and therefore QTLs seem to be everywhere in genome. Table 6 shows the largest absolute SNP effects estimated by emBayesB can explain more proportions of total additive genetic variance than that by GBLUP. We think the reason may be prior distribution of emBayesB highlights loci with large effects.

According to proportion of total additive genetic variance explained by significant SNPs (shown in Table 6), we can speculate the number of QTLs in n-3HUFA is fewer than that in BW and BL, which can offer a reference for using pre-selection of SNPs in genomic selection. Using n-3HUFA as an example, the largest absolute effects by emBayesB can explain more than 100 % of total additive genetic variance, which means the 48 SNPs is enough to perform genomic prediction in n-3HUFA. It is reasonable that a portion of SNPs explain slightly more than 100 % of total genetic variance, because not all SNP loci are associated with the trait and the SNPs with no effect may interfere with the estimation. Ulrike et al. [19] have suggested pre-selection of SNPs can improve predictive accuracies in genomic selection, which means it is possible to obtain higher additive genetic variance using a portion of SNPs.

### GS in fish breeding

Through analyzing the data in the experiment, using genome-wide markers to estimate genetic values is feasible in large yellow croaker. However, it still has some limitation to apply genomic selection in fish breeding. The reason is high mortality rate in fish, which causes genomic selection applied in fish is not the same as domestic animals completely. For example, dairy cattle and pigs can be selected in an early stage by using GEBV as a reference because they have low death rates. Most of fish, however, will die in the process of growth, which leads to the result that an early-stage selection is not a good scheme in fish breeding. If we want to select the broodfish in the early stage, more fish are required to select to meet the quantity of adult broodfish, which means more fish are required to be measured and genotyped. Therefore, the costs will increase a lot if an early-stage selection is performed in fish genomic breeding. The better way to save costs is selecting the broodfish in adult stage. In this stage, the fish have relatively low mortality, but the traits such as body size are also easy to be measured, which means phenotypic selection is viable for these traits. However, some traits such as meat quality and disease resistance are not suitable to be measured in candidates, so phenotypic selection is not suitable in these traits. Therefore, we suggest genomic selection is more suitable for some traits which cannot be measured on candidates directly. Without doubt, the selection age of fish is still in adult stage but not in early stage. In addition, because of high cost of genotyping, other methods such as pre-selection of significant SNPs [45, 46] or using extreme phenotypic records [47] can be considered in genomic prediction of fish breeding.

## Conclusions

In this study, heritability estimates by REML were 0.604, 0.586 and 0.438 for trait of body weight, body length and n-3HUFA respectively. The research showed that using genome-wide sequence data to estimate genetic values was feasible in large yellow croaker, which is helpful to promote this technology to apply in fish breeding. GBLUP and emBayesB had respective advantages on different traits, so we suggest doing a test before deciding to use which algorithm in specific trait in genomic selection. Combined with the predictive accuracy equations, we derived that at least 1000 individuals in training set could reach a predictive accuracy of 0.8 in body weight and body length. Three algorithms, i.e., GBLUP, emBayesB and GWAS, cannot always find significant SNPs associated with phenotypes consistently. The significant SNPs by emBayesB could explain the maximal proportion of total additive genetic variance, while that by GWAS explained the minimal proportion, which can offer a reference for pre-selection of SNPs in genomic selection. Because of high mortality rate of fish and high cost in genomic sequencing, genomic selection may be more suitable for applying on the traits which cannot be measured on candidates directly.

## Abbreviations

BL, body length; BLUPEBV, BLUP estimated breeding values; BLUP, best linear unbiased prediction; BW, body weight; EM, expectation-maximization; emBayesB, BayesB based on expectation-maximization; GBLUP, Genomic BLUP; GBS, Genotyping-by-sequencing; GEBV, genomic estimated breeding value; G matrix, genomic relationship matrix; GS, genomic selection; GWAS, genome-wide association study; HWE, Hardy-Weinberg equilibrium; ICE, iterated conditional expectation; LD, linkage disequilibrium; MAF, minor allele frequency; MAS, marker-assisted selection; MCMC, Monte Carlo Markov Chain; MME, mixed model equation; n-3HUFA, percentage of n-3 highly unsaturated fatty acids; NEB, New England Biolabs; QTL, quantitative trait loci; REML, Restricted Maximum Likelihood; RR-BLUP, ridge-regression BLUP; SNP, single nucleotide polymorphism; TBV, true breeding values

## References

[1] Pimentel ECG, Erbe M, Koenig S, Simianer H (2011). Genome partitioning of genetic variation for milk production and composition traits in holstein cattle. Front Genet.

[2] Weedon MN, Lango H, Lindgren CM, Wallace C, Evans DM, Mangino M (2008). Genome-wide association analysis identifies 20 loci that influence adult height. Nat Genet.

[3] Yang J, Benyamin B, McEvoy BP, Gordon S, Henders AK, Nyholt DR (2010). Common SNPs explain a large proportion of the heritability for human height. Nat Genet.

[4] Meuwissen THE, Hayes BJ, Goddard ME (2001). Prediction of total genetic value using genome-wide dense marker maps. Genetics.

[5] Goddard ME, Hayes BJ (2007). Genomic selection. J Anim Breed Genet.

[6] Schaeffer LR (2006). Strategy for applying genome-wide selection in dairy cattle. J Anim Breed Genet.

[7] Colombani C, Legarra A, Fritz S, Guillaume F, Croiseau P, Ducrocq V (2013). Application of Bayesian least absolute shrinkage and selection operator (LASSO) and BayesCπ methods for genomic selection in French Holstein and Montbéliarde breeds. J Dairy Sci.

[8] Su G, Guldbrandtsen B, Gregersen VR, Lund MS (2010). Preliminary investigation on reliability of genomic estimated breeding values in the Danish Holstein population. J Dairy Sci.

[9] VanRaden P, Sullivan P (2010). International genomic evaluation methods for dairy cattle. Genet Sel Evol.

[10] Hayes BJ, Bowman PJ, Chamberlain AJ, Goddard ME (2009). Invited review: Genomic selection in dairy cattle: Progress and challenges. J Dairy Sci.

[11] VanRaden PM, Van Tassell CP, Wiggans GR, Sonstegard TS, Schnabel RD, Taylor JF (2009). Invited Review: Reliability of genomic predictions for North American Holstein bulls. J Dairy Sci.

[12] Hayes B (2014). Genomic prediction from whole genome sequence in livestock: the 1000 bull genomes project. 10th World Congress on Genetics Applied to Livestock Production: 2014.

[13] Sun C, VanRaden PM, Cole JB, O'Connell JR (2014). Improvement of prediction ability for genomic selection of dairy cattle by including dominance effects. PLoS One.

[14] Bernardo R, Yu J (2007). Prospects for genomewide selection for quantitative traits in maize. Crop Sci.

[15] Tribout T, Larzul C, Phocas F (2012). Efficiency of genomic selection in a purebred pig male line. J Anim Sci.

[16] Christensen OF, Madsen P, Nielsen B, Ostersen T, Su G (2012). Single-step methods for genomic evaluation in pigs. Animal.

[17] Duchemin S, Colombani C, Legarra A, Baloche G, Larroque H, Astruc J-M (2012). Genomic selection in the French Lacaune dairy sheep breed. J Dairy Sci.

[18] Liu T, Qu H, Luo C, Shu D, Wang J, Lund MS (2014). Accuracy of genomic prediction for growth and carcass traits in Chinese triple-yellow chickens. BMC Genet.

[19] Ober U, Ayroles JF, Stone EA, Richards S, Zhu D, Gibbs RA (2012). Using whole-genome sequence data to predict quantitative trait phenotypes in Drosophila melanogaster. PLoS Genet.

[20] Legarra A, Robert-Granié C, Manfredi E, Elsen J-M (2008). Performance of genomic selection in mice. Genetics.

[21] Spindel J, Begum H, Akdemir D, Virk P, Collard B, Redoña E (2015). Genomic Selection and Association Mapping in rice (Oryza sativa): Effect of trait genetic architecture, training population composition, marker number and statistical model on accuracy of rice genomic selection in elite, tropical rice breeding lines. PLoS Genet.

[22] Yue GH (2014). Recent advances of genome mapping and marker‐assisted selection in aquaculture. Fish Fish.

[23] Sonesson AK, Meuwissen T (2009). Testing strategies for genomic selection in aquaculture breeding programs. Genet Sel Evol.

[24] Tsai H-Y, Hamilton A, Tinch AE, Guy DR, Gharbi K, Stear MJ (2015). Genome wide association and genomic prediction for growth traits in juvenile farmed Atlantic salmon using a high density SNP array. BMC Genomics.

[25] Wang Z, Wang Y, Lin L, Qiu S, Okamoto N (2001). Genetic polymorphisms in wild and cultured large yellow croaker Pseudosciaena crocea using AFLP fingerprinting. J Fish Sci Chin.

[26] Xiao S, Han Z, Wang P, Han F, Liu Y, Li J (2015). Functional marker detection and analysis on a comprehensive transcriptome of large yellow croaker by next generation sequencing. PLoS One.

[27] Henderson CR (1975). Best linear unbiased estimation and prediction under a selection model. Biometrics.

[28] Daetwyler HD, Villanueva B, Bijma P, Woolliams JA (2007). Inbreeding in genome‐wide selection. J Anim Breed Genet.

[29] Nielsen HM, Sonesson AK, Yazdi H, Meuwissen TH (2009). Comparison of accuracy of genome-wide and BLUP breeding value estimates in sib based aquaculture breeding schemes. Aquaculture.

[30] VanRaden P (2008). Efficient methods to compute genomic predictions. J Dairy Sci.

[31] Meuwissen T, Goddard M (2010). Accurate prediction of genetic values for complex traits by whole-genome resequencing. Genetics.

[32] Meuwissen T, Solberg TR, Shepherd R, Woolliams JA (2009). A fast algorithm for BayesB type of prediction of genome-wide estimates of genetic value. Genet Sel Evol.

[33] Shepherd RK, Meuwissen TH, Woolliams JA (2010). Genomic selection and complex trait prediction using a fast EM algorithm applied to genome-wide markers. BMC Bioinformatics.

[34] Daetwyler HD, Villanueva B, Woolliams JA (2008). Accuracy of predicting the genetic risk of disease using a genome-wide approach. PLoS One.

[35] Daetwyler HD, Pong-Wong R, Villanueva B, Woolliams JA (2010). The impact of genetic architecture on genome-wide evaluation methods. Genetics.

[36] Goddard M (2009). Genomic selection: prediction of accuracy and maximisation of long term response. Genetica.

[37] Solberg T, Sonesson A, Woolliams J (2008). Genomic selection using different marker types and densities. J Anim Sci.

[38] Yang X, Liu D, Liu F, Wu J, Zou J, Xiao X (2013). HTQC: a fast quality control toolkit for Illumina sequencing data. BMC Bioinformatics.

[39] Ao J, Mu Y, Xiang L-X, Fan D, Feng M, Zhang S (2015). Genome sequencing of the perciform fish Larimichthys crocea provides insights into molecular and genetic mechanisms of stress adaptation. PLoS Genet.

[40] Li H, Durbin R (2009). Fast and accurate short read alignment with Burrows–Wheeler transform. Bioinformatics.

[41] McKenna A, Hanna M, Banks E, Sivachenko A, Cibulskis K, Kernytsky A (2010). The Genome Analysis Toolkit: a MapReduce framework for analyzing next-generation DNA sequencing data. Genome Res.

[42] Browning BL, Browning SR (2009). A unified approach to genotype imputation and haplotype-phase inference for large data sets of trios and unrelated individuals. Am J Hum Genet.

[43] Smith S, Graser H-U (1986). Estimating variance components in a class of mixed models by restricted maximum likelihood. J Dairy Sci.

[44] Moser G, Tier B, Crump RE, Khatkar MS, Raadsma HW (2009). A comparison of five methods to predict genomic breeding values of dairy bulls from genome-wide SNP markers. Genet Sel Evol.

[45] Macciotta NP, Gaspa G, Steri R, Pieramati C, Carnier P, Dimauro C (2009). Pre-selection of most significant SNPS for the estimation of genomic breeding values. BMC proceedings: 2009.

[46] Schulz-Streeck T, Ogutu JO, Piepho H-P (2011). Pre-selection of markers for genomic selection. BMC proceedings: 2011.

[47] Yang J, Jiang H, Yeh CT, Yu J, Jeddeloh JA, Nettleton D (2015). Extreme‐phenotype genome‐wide association study (XP‐GWAS): a method for identifying trait‐associated variants by sequencing pools of individuals selected from a diversity panel. Plant J.

